# Impact of informal settlements and wastewater treatment plants on helminth egg contamination of urban rivers and risks associated with exposure

**DOI:** 10.1007/s10661-020-08660-0

**Published:** 2020-10-19

**Authors:** Isaac Dennis Amoah, Sheena Kumari, Poovendhree Reddy, Thor Axel Stenström, Faizal Bux

**Affiliations:** 1 Institute for Water and Wastewater Technology, Durban University of Technology, Durban, 4000 South Africa; 2 Department of Community Health Studies, Faculty of Health Sciences, Durban University of Technology, Durban, 4000 South Africa

**Keywords:** Helminths, Surface water, Wastewater treatment, Informal settlements, Irrigation, Risk assessment

## Abstract

The quality of surface water could be influenced by both anthropogenic and natural factors. This study was designed to determine the impact of informal settlement and wastewater treatment plants on helminth egg contamination of urban rivers and the risks associated with everyday use. We also ascertained the accumulation of these eggs in the river sediments. The study was carried out in two rivers in the eThekwini Municipality of South Africa. Grab samples were taken at different points over a 10-month period. *Ascaris* spp., hookworm, *Toxocara* spp., *Trichuris* spp. and *Taenia* spp. were the helminth eggs detected in both the water column and sediments, with mean *Ascaris* spp. eggs of 0–6.3 (± 5.1)/L in the water and 0–6.8 (± 5.2)/kg in sediment samples. The helminth egg concentrations showed seasonal variation, probably due to changes in infection levels of the populations or natural factors, such as rainfall. The informal settlements had a greater impact than treated wastewater. For every 10,000 recreational users of the rivers 19 to 58 may be infected under undisturbed conditions, increasing to 29–88 individuals when the riverbed is disturbed. The risk from agricultural use of the rivers was above the tolerable risk values applicable for wastewater reuse, recommended by the World Health Organization. This calls for a re-evaluation of the policies governing surface water quality assessment, where the inclusion of helminth eggs and sediment monitoring will be critical.

## Introduction

Surface water quality is an important factor affecting not only human health but the entire ecological system (Wang et al. 2013). This is most important in urban areas where rivers are impacted by several anthropogenic and natural factors. For example, anthropogenic activities such as industrial, agricultural and chemical spills and dam construction are major contributors to the quality of surface water (Qadir et al. 2008). These are cumulative in nature over time and space (Gazzaz et al. 2012a). Additionally, natural processes such as erosion and climatic conditions may also affect surface water quality (Zhang et al. 2010). River quality is not only an indication of the health of the river but may also reflect the health of the surrounding landscapes (Zhou et al. 2012).

Pollution from other sources such as wastewater treatment plants has an impact on river ecosystems (Bernhardt and Palmer 2007; Grant et al. 2012), affecting their everyday use. Wastewater effluents may still contain a complex mixture of contaminants such as pharmaceuticals and personal care products (Kuster et al. 2008; Ginebreda et al. 2010), microorganisms (Gazzaz et al. 2012b; Jagals 1997) and nutrients (Aristi et al. 2015). A decline in the performance of wastewater treatment plants in South Africa has been reported; in 2010, 53% of these plants were within compliance limits (Dungeni et al. 2010). However, by 2019, an average of 67% of South Africa’s sewage systems were not functioning properly (Herbig and Meissner 2019). Therefore, the discharge of effluents into surface water may result in contamination and subsequent public health issues. In addition, informal settlements, sometimes referred to as slums, may contribute to the pollution of surface water within urban areas (Abia et al. 2018; Bandyopadhyay and De 2017). The impact from these informal settlements adds to the pollution of urban surface water by wastewater treatment plant effluents.

The potential contamination of surface water from all these sources highlights the importance of water quality monitoring. However, these water quality monitoring approaches mainly focus on bacteria, such as *E. coli* and a few other coliforms (Dalla Vecchia et al. 2015; Kirschner et al. 2017; Egbueri 2019). In monitoring surface water quality, less attention has been paid to helminth egg contamination, except in instances when contamination with wastewater is suspected (Amoah et al. 2016; Fuhrimann et al. 2017). Except for wastewater reuse guidelines, no other water guideline (drinking or recreational) considers helminths as a major health threat. However, exposure to wastewater or faecally contaminated surface water has been shown to exhibit high risk of helminth infections (Amoah et al. 2016; Fuhrimann et al. 2017; Ribas et al. 2017; Mather et al. 2020). This is therefore a major gap in ensuring the protection of public health, especially in areas where helminth infections are high. For instance, although the total number of people infected with soil-transmitted helminths (STHs) in South Africa is unknown (Molvik et al. 2017), it is estimated that over 3 million children require treatment (WHO 2014). Therefore, rivers within urban areas with poor sanitation and in areas receiving wastewater effluents could be impacted by faecal contamination and in turn act as a transmission route. The risk of infection associated with wastewater or faecally contaminated wastewater during irrigation practices has been studied extensively (Fuhrimann et al. 2016; Amoah et al. 2018; Msoffe 2019); however, within urban settings, the use of the surface water goes beyond irrigation to include, potentially, recreation and other domestic uses. Additionally, the water quality monitoring relies on the water column alone without assessing the impact of river sediments (Jamieson et al. 2004; Bai and Lung 2005; Characklis et al. 2005; Fries et al. 2008). Microorganisms in the aquatic environment have the tendency to either settle, depending on their settleability, or become attached to fine suspended sediment particles (Gao et al. 2011; Abia et al. 2015). Therefore, under disturbed conditions, these may result in an increase in their concentrations in the water column leading to potentially higher risks of infections.

In the present study, we highlight the importance of the inclusion of STH analysis to water quality monitoring especially in areas with poor sanitation and wastewater effluent influence. We also show that the addition of sediment samples in the river or surface water quality monitoring may give a much more efficient estimation of potential risks. The use of the quantitative microbial risk assessment approach makes it possible to determine the potential risks of helminth infections for populations exposed to contaminated rivers. Our study therefore contributes to the design of appropriate water quality monitoring approaches and estimation of health.

## Methodology

### Study area

This study was performed in two catchments, the Isipingo and Palmiet rivers within the city of Durban, in the eThekweni municipality of South Africa. The Isipingo River is located about 20 km south of the central business area of the city and is approximately 27 km long (Pillay 2013). The Palmiet River is approximately 25 km and located within the northern periphery of the city of Durban. Table 1 presents more details about the sample points within the study areas.

**Table 1:** Description of the sampling points in the two rivers

Isipingo River	
Sampling point	Description
Pt 1	This sampling point is located 514 m upstream of a wastewater treatment plant discharge point. It was chosen to represent the water quality upstream of the discharge outflow of the wastewater treatment plant. It is also close to an informal settlement and may be influenced by its anthropogenic activities.
Pt 2	Pt 2 is located adjacent a wastewater treatment plant, downstream of Pt 1. It is impacted both by activities described for Pt 1 and by the wastewater treatment plant.
Pt 3	This point is located 1.42 km downstream of Pt 2 and is therefore downstream of the discharge point of the WWTP. This point is located close (22 m) to an informal settlement.
Pt 4	This point is at the end of the river just before joining the sea. This point is therefore located downstream of both the WWTP discharge point and the informal settlement (2.69 km). At this point, human influence is minimal.
Palmiet River	
Sampling point	Description
PA 1	This point is within an upper-class community chosen to give an indication of the water quality upstream (before) of the impact of informal settlements.
PA 2	This sample point is within the beginning of an informal settlement located along this river and 1.54 km from PA 1.
PA 3	This sample point is located in the middle of the informal settlement and 302 m from PA 2.
PA 4	The Palmiet River joins one of Durban’s largest rivers, the Umgeni, at this point, which is considered to be downstream of the informal settlement. It is approximately 1 km from PA 3.

### Sampling

Surface water and sediment samples were collected monthly from January to October 2016. Grab samples were taken in triplicates of 1 L using sterilized containers, approximately 0.5 m below the water surface at each sampling point. Care was taken not to disturb the sediments. Sediment sampling was adapted from Adeyinka et al. (2019); briefly, composite sediment samples were taken from the top 5 cm at each point by using a hand-held spade to collect the sediments into a 500-mL bucket.

### Laboratory analysis

The water samples were analysed for helminth eggs using a modified method based on the principle of centrifugation and flotation presented in Amoah et al. (2018). Only viable helminth eggs determined using the method referenced were counted and reported in this paper. All the pellets incubated were viewed under the microscope (×100), counted and reported per 1 kg for the sediments and 1 L for the water samples.

### Statistical analysis

The concentration of eggs at the different sampling points and sites was described through descriptive statistics using Excel (2016 version, Microsoft Corporation). To determine the statistical significance or otherwise, difference in concentration of the eggs at the sampling points was determined using the Kruskal-Wallis tests and the Mann-Whitney *U* test used to compare the concentrations between the helminth eggs in the water column and the sediments using a 95% confidence interval (Bethea et al. 1995). Additionally, seasonal variation was determined by comparing the concentrations of the different helminth eggs over the four seasons prevalent in South Africa, thus autumn: March–May, winter: June–August, spring: September–October and summer: January–February. This was performed using the Kruskal-Wallis tests with Dunn’s multiple-comparison test afterwards. All statistical analysis was performed in Graphpad Prism 7 software (GraphPad Software, Inc. USA).

### Risk assessment

The quantitative microbial risk assessment (QMRA) approach was used to assess the risks of helminth infections as described by the four steps below:

#### Hazard identification

For the purposes of QMRA, only *Ascaris* spp. have a dose-response model, which was therefore chosen as the index for the helminths.

#### Exposure assessment

In this study, three exposure scenarios were considered; exposure during recreation, irrigation and indirectly through consumption of irrigated vegetables.

#### Dose-response assessment

The exponential dose-response model (Westrell 2004; Seidu et al. 2008) given by the formula below was used:

$$ {P}_{\mathrm{inf}}=1-{e}^{- rd} $$

where *P*_inf_ is the probability of infection associated with the ingestion of *Ascaris* spp. eggs, *r* the dimensionless infectivity constant for *Ascaris* spp. and *d* the dose of the eggs ingested under each scenario considered. An *r* value of 0.039 was used in this assessment (Navarro et al. 2008). The dose of *Ascaris* spp. eggs ingested per exposure was modelled by fitting a probability distribution function to the concentrations reported in this study. Increase in *Ascaris* spp. concentration in the water column from disturbance was also considered where it was assumed that concentrations will increase by 30–55% (Krometis et al. 2007).

The dose of *Ascaris* spp. eggs ingested during recreational use or during irrigation of crops was determined using the formula:

$$ D={C}_{\mathrm{raw}}\times V $$

where *D* is the concentration (dose) ingested by the swimmer or farmer, *C*_raw_ the concentration of *Ascaris* spp. eggs per millilitre and *V* the volume (mL/day) ingested by swimmer or farmer. The dose (*D*_C_) of *Ascaris* spp. eggs ingested by consumers was modelled with lettuce as a surrogate vegetable using the formula

$$ {D}_{\mathrm{C}}= Vlc $$

where *V* is the volume of water caught on the lettuce in millilitres per gram of lettuce, *I* the mean per capita intake of lettuce in grams per person per day and *c* the concentration of *Ascaris* spp. eggs in the water used for irrigation. The different exposure scenarios and volumes ingested are presented in Table 2.

**Table 2:** Assumptions used in estimation of risks of *Ascaris* spp. infections for exposed different groups

Exposure scenario/assumptions for dosage	Volume of water ingested (ml or g)	Frequency (days)	Reference
Ingestion by swimmers	Uniform distribution (10, 15)	Uniform distribution (64,128)	Dorevitch et al. 2011; Amoah et al. 2018
Ingestion by farmers	Uniform distribution (1–5)	Uniform distribution (120, 140)	WHO 2006; Amoah et al. 2018
Consumption of lettuce		Uniform distribution (156,160)	Amoah et al. 2018
Volume of water caught on lettuce	Normal distribution (0.108, 0.019)		Hamilton et al. 2006
Per capita intake of lettuce	Pert distribution (25, 50, 75)		Sant’Ana et al. 2014

#### Risk characterization

Risks of infection from multiple exposures were determined using the formula;

$$ {\mathrm{P}}_1\left(\mathrm{A}\right)=1-{\left(1-{\mathrm{P}}_1\left(\mathrm{d}\right)\right)}^{\mathrm{n}} $$

where P_1_(A) is the risk of infection after multiple exposures, *P*_*1*_(*d*) the risk of infection from a single exposure to a dose *d* of the *Ascaris* spp. egg and *n* is the number of days of exposure to the single dose *d* (Sakaji and Funamizu 1998).

## Results

### Concentration of helminth eggs in water and sediments

Eggs of *Ascaris* spp., hookworm, *Toxocara* spp., *Trichuris* spp. and *Taenia* spp. were detected in both the water and sediments, with *Ascaris* spp. and hookworm the most abundant. In the Isipingo River, *Ascaris* spp. eggs ranged from 0 to 6.3 (± 5.1)/L in the water and 0–6.8 (± 5.2)/kg in the sediments. Similarly, for hookworm eggs, high concentrations were found in the sediments (0–6.6 (± 5.7)/L) (Table 3). The highest concentration of eggs was found at the sampling point next to the transit camp (point 3) both for the water and sediment samples. These differences in egg concentrations were statistically significant (*p* value ≤ 0.05).

**Table 3:** Mean concentration (± SD) of helminth eggs in water (per litre) and sediment (per kg) at various sampling points in the Isipingo River

	Upstream of WWTP discharge point (Pt 1)		Next to WWTP discharge point (Pt 2)		Next to transit camp, downstream of WWTP (Pt 3)		Joining the sea (Pt 4)	
	Water	Sediments	Water	Sediments	Water	Sediments	Water	Sediments
*Ascaris* spp.	1 (± 1.7)	1.6 (± 2.6)	3.4 (± 3.8)	3.6 (± 3.9)	6.3 (± 5.1)	6.8 (± 5.2)	0	0
Hookworm	1.4 (± 2.3)	1.8 (± 2.6)	1.4 (± 2.9)	2 (± 3.3)	5.3 (± 3.7)	6.6 (± 5.7)	0	0
*Toxocara* spp.	1 (± 1.9)	1.8 (± 2.9)	2.2 (± 2.6)	2.6 (± 2.3)	3.4 (± 2.9)	3.8 (± 3.2)	0	0
*Trichuris* spp.	0 (± 0)	0 (± 0)	1.2 (± 1.9)	2.2 (± 2.9)	3.5 (± 3.4)	4.6 (± 5.2)	0	0
*Taenia* spp.	0.4 (± 1.3)	1.2 (± 2.2)	2.6 (± 2.8)	1.9 (± 2.8)	3.6 (± 2.8)	4.6 (± 3.6)	0	0

The occurrence of helminth eggs was similar both in relation to speciation and abundance in the water and sediments from Palmiet River. Mean *Ascaris* spp. eggs was 10 (± 8.4)/L and 12.9 (± 8.2)/kg in the water and sediments respectively. *Taenia* spp. were less abundant than others in the water samples (4.0 (± 3.5)/L), and *Toxocara* spp. eggs in the sediment samples had a mean concentration of 4.5 (± 3.1)/kg. These differences were not statistically significant (*p* value ≥ 0.05). The sampling point at the beginning of the informal settlement (PA 2) recorded the highest egg concentration. For example, the mean *Ascaris* spp. egg concentration in the water from PA 2 was 10 (± 8.5)/L while corresponding counts were 3.4 (± 3.4)/L for the sampling point where the Palmiet River joins the Umgeni River (PA 4) (Table 4). The difference in helminth egg concentrations at the various sampling points was statistically significant (*p* value ≤ 0.05).

**Table 4:** Mean concentration (± SD) of helminth eggs in water (per litre) and sediment (per kg) at various sampling points in the Palmiet River

	Upstream of informal settlement (PA 1)		Beginning of informal settlement (PA 2)		Middle of informal settlement (PA 3)		Joining the Umgeni River (PA 4)	
	Water	Sediments	Water	Sediments	Water	Sediments	Water	Sediments
*Ascaris* spp.	0	0	10 (± 8.4)	12.9 (± 8.2)	8.3 (± 5.7)	10.9 (± 7.2)	3.4 (± 3.4)	5.3 (± 3.1)
Hookworm	0	0	6.5 (± 4.8)	10.2 (± 6.8)	3.8 (± 3.8)	4.7 (± 4.9)	2 (± 2.9)	2.6 (± 2.8)
*Toxocara* spp.	0	0	5.2 (± 3.3)	4.5 (± 3.1)	5.2 (± 3.3)	4 (± 4.4)	3.4 (± 3.1)	4.9 (± 3.7)
*Trichuris* spp.	0	0	4.9 (± 3.9)	7.9 (± 4.8)	5.9 (± 3.2)	6.2 (± 3.9)	5 (± 3.6)	3.2 (± 3.2)
*Taenia* spp.	0	0	4 (± 3.5)	6.2 (± 3.5)	4.6 (± 3.4)	6.4 (± 5.2)	3.7 (± 3.3)	5.4 (± 3.7)

### Variation in helminth egg concentration over the study period

In the Isipingo River, mean *Ascaris* spp. egg concentrations in the water increased steadily from February to April, then dropped to the lowest of 0.5 (± 1)/L in June. Then again, from a mean concentration of 1.5 (± 3)/L in July, the concentration increased to 4 (± 4.8)/L in October. In contrast, *Ascaris* spp. eggs in the sediments saw a steady increase from 0 in March to 7 (± 5.8)/kg in June and declined to 0 in October. Similar trends were found for the other helminths. These variations in the concentrations were statistically significant (*p* value ≤ 0.05).

There was an observed difference in helminth egg concentrations considering the different seasons within the study area. In the water samples from the Isipingo River, the observed difference was statistically significant (*p* value ≤ 0.05). Spring had the highest concentrations for almost all the helminths identified; *Ascaris* spp. (3.9 ± 0.2 eggs/L), hookworm (3.4 ± 1.2 eggs/L), *Trichuris* spp. (2.8 ± 0.4 eggs/L) and *Taenia* spp. (3.9 ± 0.2 eggs/L). The only exception was observed for *Toxocara* spp., where the highest concentrations were observed in summer (3 ± 0.7 eggs/L). However, the difference in egg concentrations in the sediments did not exhibit any statistically significant differences. No one season stood out in terms of egg concentrations.

**Fig. 1:**
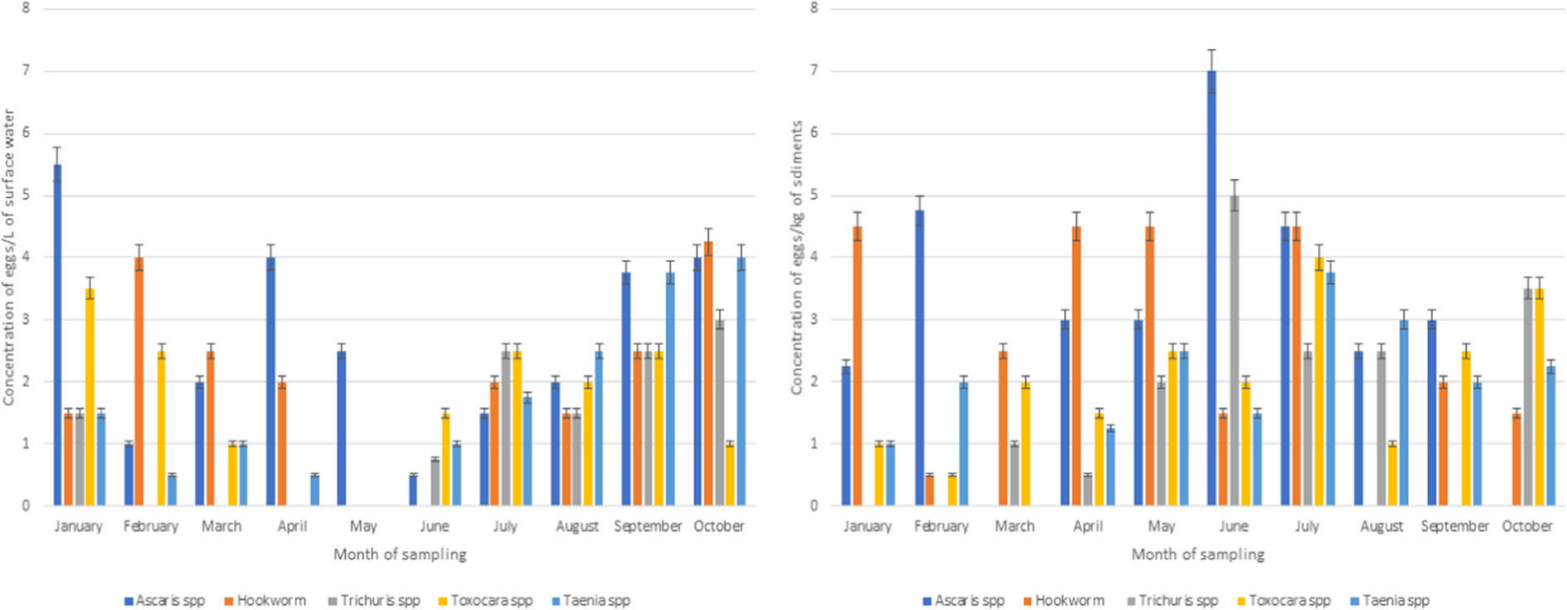
Concentration of helminth eggs in surface water and sediments in the Isipingo River

**Fig. 2:**
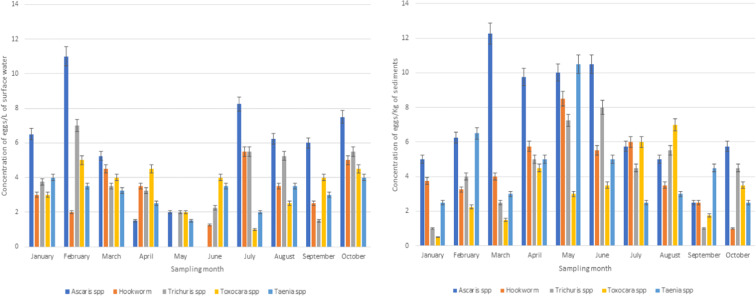
Concentration of helminth eggs in surface water and sediments in the Palmiet River

In the Palmiet River, the differences in the concentrations over the months were much clearer than in the Isipingo River. For instance, as shown in Figs. 1 and 2, the concentrations of *Ascaris* spp., hookworm and *Trichuris* spp. eggs (respectively) were higher in the months of March to June. In addition, the overall concentration of these eggs in the Palmiet River was higher than in the Isipingo River as can be seen by comparing results in Figs. 1 and 2. Water samples from the Palmiet River did not show any statistical difference in relation to seasonal variation in egg concentrations. However, mean egg concentrations were higher in summer for *Ascaris* spp. (8.8 ± 3.2 eggs/L), *Trichuris* spp. (5.4 ± 2.3 eggs/L) and *Taenia* spp. (3.8 ± 0.4 eggs/L). Hookworm and *Toxocara* spp. concentrations were highest in spring (3.8 ± 1.8 and 4.3 ± 0.4 eggs/L respectively). However, in the sediment samples analysed, the differences in egg concentrations between the four seasons were statistically significant (*p* value ≤ 0.05). In these sediments, the highest concentrations were observed for the seasons of autumn (*Ascaris* spp. (10.7 ± 1.4 eggs/kg), hookworm (6.1 ± 2.3 eggs/kg) and *Taenia* spp. (6.2 ± 3.9 eggs/kg)) and winter (*Trichuris* spp. (6.0 ± 1.8 eggs/kg) and *Toxocara* spp. (5.5 ± 1.8 eggs/kg)).

### Risk of *Ascaris* spp. infection after ingestion of eggs

Infection with helminths is associated with exposure to the water either through intentional or accidental ingestion. Recreational use (swimming or bathing) of the Palmiet River may result in median risks of 19 to 58 out of 10,000 people being infected under normal conditions. However, with disturbance of the sediments, the risks increased to between 29 to 88 out of 10,000; the difference in risk from under the normal and disturbed conditions was statistically significant (*p* value ≤ 0.05). The highest risks as expected were observed at points with high contamination as described above (Table 5). The risks of *Ascaris* spp. infections for swimmers at the Isipingo River were much lower than those for the Palmiet River. For one-time exposures, the risks were in the range from 2 out of 100, 000 to 33 out of 10,000 under normal conditions and 6 out of 100,000 to 50 out of 10,000 when the sediments are disturbed with increase for annual exposures (Table 5).

**Table 5:** Risks of *Ascaris* spp. infections for recreational use of the two rivers

Palmiet River			Isipingo River		
Sampling points	Risks of infection under normal water conditions (± SD)	Risks of infection under disturbed conditions (± SD)	Sampling points	Risks of infection under normal water conditions (± SD)	Risks of infection under disturbed conditions (± SD)
Beginning of informal settlement (PA 2)	1.2 × 10^−3^ (± 1.96 × 10^−5^)	1.8 × 10^−3^(± 6.29 × 10^−5^)	Upstream of WWTP discharge point (Pt 1)	6.1 × 10^−5^ (± 2.18 × 10^−6^)	1.1 × 10^−3^ (± 1.37 × 10^−5^)
Middle of informal settlement (PA 3)	7.8 × 10^−4^(± 1.30 × 10^−5^)	1.4 × 10^−3^(± 1.74 × 10^−5^)	Next to WWTP discharge point (Pt 2)	2.1 × 10^−4^ (± 7.23 × 10^−6^)	3.9 × 10^−4^ (± 8.58 × 10^−6^)
Joining the Umgeni River (PA 4)	3.9 × 10^−4^(± 6.54 × 10^−5^)	6.3 × 10^−4^(± 8.21 × 10^−5^)	Next to Transit camp (downstream of WWTP) (Pt 3)	6.8 × 10^−4^ (± 1.14 × 10^−5^)	1.4 × 10^−4^ (± 2.94 × 10^−6^)

Similar difference in risks between the two rivers was observed for irrigational use of the water by farmers; in the Palmiet River, one time use of the water results in risks of infections ranging from 4 to 12 out of 10,000 farmers infected under normal water conditions, with an increase when the sediments are disturbed (Table 6).

**Table 6:** Risks of *Ascaris* spp. infections for farmers using the two rivers for irrigation

Palmiet River			Isipingo River		
Sampling points	Risks of infection under normal water conditions (± SD)	Risks of infection under disturbed conditions (± SD)	Sampling points	Risks of infection under normal water conditions (± SD)	Risks of infection under disturbed conditions (± SD)
Beginning of informal settlement (PA 2)	5.8 × 10^−3^ (± 6.89 × 10^−5^)	8.8 × 10^−3^ (± 0.0001943)	Upstream of WWTP discharge point (Pt 1)	2.9 × 10^−4^ (± 8.11 × 10^−5^)	5.0 × 10^−3^ (± 4.58 × 10^−5^)
Middle of informal settlement(PA 3)	3.8 × 10^−3^ (± 4.59 × 10^−5^)	6.5 × 10^−3^ (± 5.67 × 10^−5^)	Next to WWTP discharge point (Pt 2)	9.7 × 10^−4^ (± 2.75 × 10^−5^)	1.8 × 10^−3^ (± 3.12 × 10^−5^)
Joining the Umgeni River (PA 4)	1.9 × 10^−3^ (± 2.30 × 10^−5^)	2.9 × 10^−3^ (± 2.68 × 10^−5^)	Next to transit camp (downstream of WWTP) (Pt 3)	3.3 × 10^−3^ (± 4.03 × 10^−5^)	6.4 × 10^−4^ (± 1.06 × 10^−5^)

Consumption of lettuce irrigated with water from the Palmiet River may lead to 8 out of 10,000 to 23 out of 10,000 of the consumers infected under normal water conditions. Corresponding risks from the Isipingo River were 11 out of 100,000 to 14 out of 10,000 consumers (Table 7). Annual consumption of the lettuce increases the risks significantly.

**Table 7:** Risks of *Ascaris* spp. infections for consumers of vegetables irrigated with water from the two rivers

Palmiet River			Isipingo River		
Sampling points	Risks of infection under normal water conditions (± SD)	Risks of infection under disturbed conditions (± SD)	Sampling points	Risks of infection under normal water conditions (± SD)	Risks of infection under disturbed conditions (± SD)
Beginning of informal settlement (PA 2)	2.3 × 10^−3^ (± 3.19 × 10^−5^)	3.6 × 10^−3^ (± 8.99 × 10^−5^)	Upstream of WWTP discharge point (Pt 1)	1.2 × 10^−4^ (± 3.66 × 10^−6^)	2.1 × 10^−3^ (± 2.16 × 10^−5^)
Middle of informal settlement (PA 3)	1.6 × 10^−3^ (± 2.13 × 10^−5^)	2.7 × 10^−3^ (± 2.71 × 10^−5^)	Next to WWTP discharge point (Pt 2)	4.0 × 10^−4^ (± 1.27 × 10^−5^)	7.5 × 10^−4^ (± 1.44 × 10^−5^)
Joining the Umgeni River (PA 4)	7.8 × 10^−4^ (± 1.06 × 10^−5^)	1.2 × 10^−3^ (± 1.27 × 10^−5^)	Next to transit camp (downstream of WWTP) (Pt 3)	1.4 × 10^−3^ (± 1.85 × 10^−5^)	2.7 × 10^−4^ (± 4.84 × 10^−6^)

## Discussion

The dominance of *Ascaris* spp. and hookworm eggs in the water and sediments could be attributed to the human infections reported in South Africa (Appleton et al. 2009; Mkhize-Kwitshana and Mabaso 2014; Molvik et al. 2017). Additionally, our previous studies in the area gave a similar profile of helminth eggs in wastewater and sludge (Amoah et al. 2018). Therefore, the type and concentration of the helminths reported in this study are corroborated by these reports from clinical infections and wastewater/sludge analysis. The concentrations in the water column could also be influenced by natural events such as rainfall. For instance, rainfall may result in the increase of pathogen concentrations in water columns due to resuspension of the sediments (Muirhead et al. 2004; Griffith et al. 2010). Krometis et al. (2007) reported an increase by 30–55% of the presence of different indicator organisms under disturbed conditions. With settling velocities of 0.65 m/h for *Ascaris* spp. eggs and 1.53 m/h for *Trichuris* spp. eggs (David and Lindquist 1982; Dryden et al. 2005), most of these eggs may be in the sediments under normal river conditions with an expected increase in the water column following sediment disturbance. This could account for the significantly higher egg concentrations in the sediments from the Palmiet River than the water, for *Ascaris* spp., hookworm and *Taenia* spp., which may be due to the impact of the informal settlements in this study area.

The difference in the helminth egg concentration at the different sampling points could be attributed to the various activities along these rivers. For instance, in the Palmiet River, the highest concentrations were found at points directly influenced by informal settlements. Collectively, the two sampling points within the informal settlements had an average of 5.8 (± 1.9) eggs/L and 7.4 (± 3.0) eggs/kg for water and sediments respectively. Comparatively, the first sampling point (PA 1) had no helminth eggs, probably due to the absence of direct human contact or impact with the river at this section. PA 4 had low concentration (3.5 (± 1.1) eggs/L for water and 4.3 (± 1.3) eggs/kg for the sediments) compared to the two points within the settlement. This sampling point is located about 1 km away from the centre of the settlement (downstream) and may be the reason for the lesser helminth egg concentrations. Helminth infection is strongly correlated with the socio-economic status of the population (Stolk et al. 2016). Inhabitants of these informal settlements are usually migrants who moved to the cities in search of jobs; they live in make-shift accommodations. Therefore, these informal settlements are characterized by poor socio-economic and housing conditions with poor sanitation. Linked with the lack of proper sanitation, some of the inhabitants especially the children (who are the most vulnerable group) defecate near these rivers contributing to the high helminth egg concentrations reported at these points. The impact of open defecation on surface water contamination has been observed by other studies (Semwal and Akolkar 2006; Vijay et al. 2011). The impact of the informal settlements on the river water quality was higher than that of the wastewater treatment although these were not on the same river. Based on calculations, the wastewater effluents contributed 2.3 (± 0.4) eggs/L and 2.8 (± 0.6) eggs/kg for the water and sediments, respectively, to the concentration of the helminth eggs in the river. Additionally, well-functioning wastewater treatment plants are expected to reduce the concentration of these parasites as well as other pathogens before discharge, which may have also contributed to the lesser impact from these plants in the study. For instance, in our assessment of the wastewater treatment plant discharging into the Isipingo River, we observed removal efficiency between 72 and 100% for helminth eggs (Amoah et al. 2018). Therefore, the influence will be lesser compared to the informal settlements where open defecation may result in the direct deposition of the eggs into the river without treatment. A study in Argentina reported an average *A. lumbricoides* concentration of 5 eggs/L in the Arias-Arenales River (Kundu et al. 2014). The concentrations reported in some of the sections of the two rivers we studied were similar to the Argentinian study; however, concentrations in the Palmiet River were higher especially within the informal settlement than the report from Argentina.

The variations in the helminth egg concentration over the 10-month study period may just reflect a normal variation between grab samples or be influenced by environmental factors. For instance, during the months of May–July, the concentrations were higher in the sediments than the surface water. These months are characterized by lower rainfall levels, resulting in slower flow rate of the rivers which may aid egg settling. With rainfall, the flow rate increases as well as dislodgement of the eggs into the water column. This was seen in both rivers, but more evident in the Palmiet River, which is the most influenced by an informal settlement. Seasonality has been associated with different diseases (Pafčo et al. 2017; Martinez 2018; Mayengue et al. 2020; Poulin 2020). It has been reported that each disease has its own window of occurrence which may vary from one geographical location to another (Martinez 2018). In this study, the observed seasonal variation in helminth egg concentrations, although not statistically significant in some instances, may be an indication of this seasonal dependent infection dynamics. The infection dynamics could be as a result of an increased exposure to these parasites in the water, resulting in infections, or it could be that the increased infections resulted in increased occurrence in the rivers. Considering that most of these are STHs that require the soil in their life cycle to become infectious, the former scenario (increased exposure) could be the most likely reason. Although there is lack of information on seasonal impact on helminth infections in humans, in sheep and other livestock, an increase in intestinal nematode infections has been observed (Waller et al. 2004; Ahmed 2017).

The determined risks from recreational use of these rivers resulted in lower likelihood of ascariasis as expected. For instance, 19 to 58 and a maximum of 33 people out of 10,000 are at risk of infection for the recreational use of the Palmiet and Isipingo rivers, respectively. Although these numbers are low, this reflects no disturbance conditions and therefore does not factor in the increase in egg concentrations in the surface water during events such as storms and rainfall. With appropriate incorporation of the effect of disturbance, these risks increase to levels that call for public health concern (refer to the section on “Risk of *Ascaris* spp. infection after ingestion of eggs”). Annual exposure to the water will result in increased risks of infection (Tables 5, 6, and 7), therefore creating major public health concerns. There is a lack of studies on the risks of helminth infections from recreational use of contaminated rivers. However, the Argentinian study referenced earlier (Kundu et al. 2014) reported that accidental ingestion of water during recreation by children resulted in risks of 1.31 × 10^−4^; in adults the risks were lower (6.47 × 10^−5^), as well as secondary recreators (6.50 × 10^−6^). Therefore, our risk estimates from recreational exposure are corroborated by that study.

In contrast to recreational use, a lot of attention has been placed on the risks of helminth infections due to agricultural use of wastewater or faecally contaminated surface water. To protect public health, the WHO recommended that wastewater used for unrestricted agriculture should have ≤ 1 helminth egg per litre (WHO 2006). However, the surface water which is influenced by wastewater contains eggs above this recommended levels. Therefore, we observed a high risk of infection for farmers using the Palmiet River, which was higher than the tolerable risk (10^−3^ per person per year) value recommended by the WHO (Mara et al. 2007), as well as risks estimated for the Arias-Arenales River in Argentina (10^−4^). However, the risks of infections for the farmers using the Isipingo River were much lower than the tolerable risk values from WHO. Similar risks were observed for consumers of lettuce irrigated with the river water, also higher than the WHO tolerable risks figures for consumers (WHO 2006) when the Palmiet River is considered. This was determined with the assumption that no further reduction in concentrations will occur from the point of harvest to consumption. However, Amoah et al. (2011) reported that washing or disinfection of the vegetables with bleach or vinegar could potentially reduce risks of infections. In addition, cessation of irrigation for some days before harvesting has been recommended (Keraita et al. 2007). However, this approach may not be applicable, especially under dry conditions, where without irrigation for a few days, produce loss may occur. Several studies have looked at the risks of helminth infections for farmers and consumers using wastewater (Barker et al. 2014; Seidu et al. 2008). The reports from these studies indicate that the concentration of the helminth eggs in the irrigation water is the key factor in ascertaining the level of risks. These were the considerations used in developing the WHO wastewater reuse guidelines mentioned above. Additionally, these eggs may accumulate in the soil after each irrigation activity (Seidu et al. 2008) and survive for longer periods of time (Zdybel et al. 2015: Gaspard et al. 1995), increasing the risks of infection further.

## Conclusion

The microbial quality of the two rivers studied was found to be poor with high concentrations of helminth eggs, especially in the sediments. It is therefore important that assessment of river water quality includes sediment analysis to give a better assessment. The high concentration of the helminth eggs highlight the importance of helminth analysis in addition to the routine indicator organism analysed. This is especially very critical in urban settings where faecal contamination of surface water is common, as shown in this study. This is because daily use of these urban rivers may increase helminth infections in these areas for exposed populations. Additionally, there has been a lot of focus on the role wastewater treatment plants play in surface water contamination; however, our study has shown that informal settlements or slums with poor sanitation may have a much higher impact. It is therefore imperative that for a long-term solution to urban pollution of rivers, we should also focus on improving sanitation coverage in our inner cities, especially slums and informal settlements, in addition to improvements in wastewater treatment.
